# Engineering a recombination-resistant live attenuated vaccine candidate with suppressed interferon antagonists for PEDV

**DOI:** 10.1128/jvi.00451-25

**Published:** 2025-06-12

**Authors:** Mingde Liu, Bikash Aryal, Xiaoyu Niu, Qiuhong Wang

**Affiliations:** 1 Center for Food Animal Health, Department of Animal Sciences, College of Food, Agricultural and Environmental Sciences, The Ohio State University 462953 , Wooster, Ohio, USA; 2 Department of Veterinary Preventive Medicine, College of Veterinary Medicine, The Ohio State University 70728, Columbus, Ohio, USA; Loyola University Chicago - Health Sciences Campus, Maywood, Illinois, USA

**Keywords:** porcine epidemic diarrhea virus, PEDV, coronavirus, live attenuated vaccine, recombination resistance, interferon antagonist, IFN, transcriptional regulatory sequence, TRS

## Abstract

Porcine epidemic diarrhea virus (PEDV) is a deadly coronavirus (CoV) for neonatal piglets, and no effective vaccine is available. Live attenuated vaccines (LAVs) show promise, but risks of recombination and reversion to virulence hinder their application. For LAV development, we previously engineered a recombination-resistant PEDV mutant RMT by rewiring the transcriptional regulatory sequence (TRS)-core sequences (TRS-CSs). Because TRS is critical for CoV replication, the incompatibility between wild-type TRS-CS and the rewired TRS-CS should disrupt the structural and accessory protein-encoding mRNA transcription from a recombinant PEDV genome, preventing the production of progeny infectious viruses. However, the RMT contained a 189-nt insertion upstream of the E gene TRS-CS and a missing guanine in the N gene TRS-CS. Here, we generated RMTv1 via correcting these mutations and removing the EGFP gene in RMT. Using the RMTv1 as a backbone, we generated a series of PEDV mutants carrying one or two attenuating mutations of non-structural protein 1 (nsp1), nsp15, and nsp16. Their replication efficiency, sensitivity to interferons (IFNs), induction of IFNs, and genetic stability were tested in Vero and/or LLC-PK1 cells. We selected RMTv1 and RMTv1-nsp1 + nsp15 for the pathogenesis studies in neonatal gnotobiotic pigs and tested the immunogenicity of RMTv1-nsp1 + nsp15. The RMTv1-nsp1 + nsp15 was further attenuated, causing no pig mortality, compared with RMTv1. All the pigs infected with RMTv1-nsp1 + nsp15 were protected from severe diarrhea and death post-challenge with virulent PEDV at 21 days post-inoculation, whereas 50% of mock-challenged piglets died. These findings establish RMTv1-nsp1 + nsp15 as a promising PEDV LAV candidate and can be further evaluated.

**IMPORTANCE:**

PEDV continues to cause devastating economic losses in the global swine industry. Exposing pregnant sows to feedback materials from infected pigs is still one of the main methods used to control PEDV outbreaks in U.S. swine farms but carries the risk of transmitting other pathogens. Effective and safe vaccines are desperately needed to replace the feedback materials but are still not available. We revised the recombination-resistant vaccine backbone and combined it with targeted attenuation mutations in viral IFN antagonists to generate six PEDV mutants. Among them, the RMTv1-nsp1 + nsp15 had significant advancements in safety and protective efficacy in neonatal piglets, demonstrating its vaccine potential to control PEDV outbreaks and improve swine health globally. By addressing key challenges in LAV development, including risks of reversion to virulence and generation of new variants via recombination, this work establishes a robust foundation for PEDV vaccine strategies and potentially inspires the development of vaccines against other emerging coronaviruses.

## INTRODUCTION

Porcine epidemic diarrhea is a highly contagious intestinal disease in swine caused by porcine epidemic diarrhea virus (PEDV), with typical clinical signs including diarrhea, vomiting, and dehydration, leading to up to 100% mortality in suckling piglets (1–4). In 2013, highly virulent PEDV variants emerged in the United States, resulting in the death of 7 million piglets within the first year of outbreaks and inflicting economic losses ranging from $900 million to $1.8 billion on the American swine industry (5, 6). Despite sporadic outbreaks in recent years, PEDV continues to pose a threat to certain U.S. pig farms, as effective vaccines remain unavailable for PEDV-naive pigs (7). Furthermore, PEDV has become the second most impactful pathogen for swine in China, home to over 50% of the global swine population (8–10). In the winter of 2020, a new wave of PEDV epidemic swept across China (11–13). Hence, there is an urgent need for the development of effective and safe vaccines against highly virulent PEDV strains.

PEDV mainly kills the suckling piglets, but the rapid onset of the disease in newborns leaves insufficient time for vaccination to induce protective immunity. Consequently, they depend on the maternal antibodies in the colostrum and milk from immunized sows/gilts to defend against PEDV during early life (14, 15). Compared to killed or spike (S) protein-based vaccines, evidence increasingly shows that the viral replication in sow enterocytes, stimulating mucosal immune responses, is crucial for the gut–mammary gland–secretory IgA axis, thereby inducing stronger and longer-lasting maternal immunity to safeguard piglets from virulent PEDV infection (16–20). Therefore, immunization of pregnant sows or gilts with feedback materials from infected pigs followed by boosting with the commercial S protein-based vaccine (Merck & Co.), or the inactivated vaccine (Zoetis Inc.) is used to control PEDV outbreaks on U.S. swine farms. However, this practice poses a risk of transmitting other pathogens from the feedback materials. Additionally, it is challenging to accurately control the viral dosage in feedback materials, making it difficult to ensure the safety and efficacy of each administration. Consequently, live attenuated vaccines (LAVs) inducing adequate maternal immunity are urgently needed to replace the feedback materials (21–25).

However, coronaviruses (CoVs) exhibit a high frequency of recombination during replication, potentially altering virulence and posing risks to the emergence of new variants (26–42). The potential recombination and mutation in LAVs raise concerns about reversion to virulent strains and generation of new variants, which can lead to vaccination failure and complicate PEDV surveillance. To mitigate these risks, the strategy of remodeling transcriptional regulatory sequences (TRS) has been proposed (43, 44). During coronavirus replication, the TRS elements are critical for the discontinuous transcription process, facilitating the production of negative-sense subgenomic RNAs (sgRNAs) that are later used as templates for the synthesis of subgenomic messenger RNAs (sgmRNAs) required for the translation of structural and accessory proteins (45–50). The TRS elements consist of the leader TRS (TRS-L) located in the 5′ untranslated region (UTR) and body TRSs (TRS-Bs) positioned upstream of genes encoding S, open reading frame 3 (ORF3), envelope (E), membrane, and nucleocapsid (N) proteins. During the synthesis of negative-sense sgRNAs by the viral RNA-dependent RNA polymerase (RdRp, nsp12), TRS-B acts as a “pause” signal. At this point, RdRp can either continue transcription to generate a longer RNA molecule or switch template to the TRS-L, producing a shorter sgRNA. The template switching at TRS-B is guided by complementary base pairing between the negative-sense TRS-B of the nascent sgRNA and the positive-sense TRS-L. Within the TRS region, a conserved core sequence (CS) consisting of 6–8 nucleotides (nt) is flanked by variable sequences at its 5′- and 3′-ends. To avoid recombination events between PEDV LAVs and field strains, we previously designed a recombination-resistant PEDV vaccine platform, designated RMT, by remodeling the TRS-CS (43). The rewired TRS-CS was incompatible with the wild-type TRS-CS, resulting in no recombinant virus particles being generated in theory. However, the RMT mutant contained two unexpected mutations: a 189-nt insertion upstream of the E gene TRS-CS and a missing guanine (G) in the N gene TRS-CS.

Except for the TRS-CS remodeling, introducing additional distantly separate attenuation mutations can further improve the safety of the LAV candidate because it requires more mutations to revert to virulence. Non-structural proteins nsp1 (51), nsp15 (52), and nsp16 (53) of PEDV are known interferon (IFN) antagonists. These proteins play crucial roles in evading the host’s immune response, thereby enhancing viral replication and pathogenicity. Mutations suppressing these IFN antagonists can significantly attenuate the virus, making them ideal targets for the development of safer and more effective LAVs (54).

In this study, we developed a revised recombination-resistant PEDV LAV backbone, RMTv1, by correcting unexpected mutations and removing the EGFP gene to leave space for expressing other peptide/protein genes in the future. This backbone represents significant progress and benefits future PEDV LAV development. Further attenuation was achieved by suppressing one or the combinations of two IFN antagonists nsp1, nsp15, and/or nsp16. The replication efficiency, IFN induction, sensitivity to IFN pre-treatment, and genetic stability of the targeted mutations were assessed *in vitro*. The best vaccine candidate was selected for *in vivo* evaluation in neonatal gnotobiotic (Gn) piglets, along with its backbone RMTv1.

## RESULTS

### Revised recombination-resistance vaccine backbone RMTv1 was rescued, and interferon antagonist mutations were introduced into RMTv1

**Fig 1:**
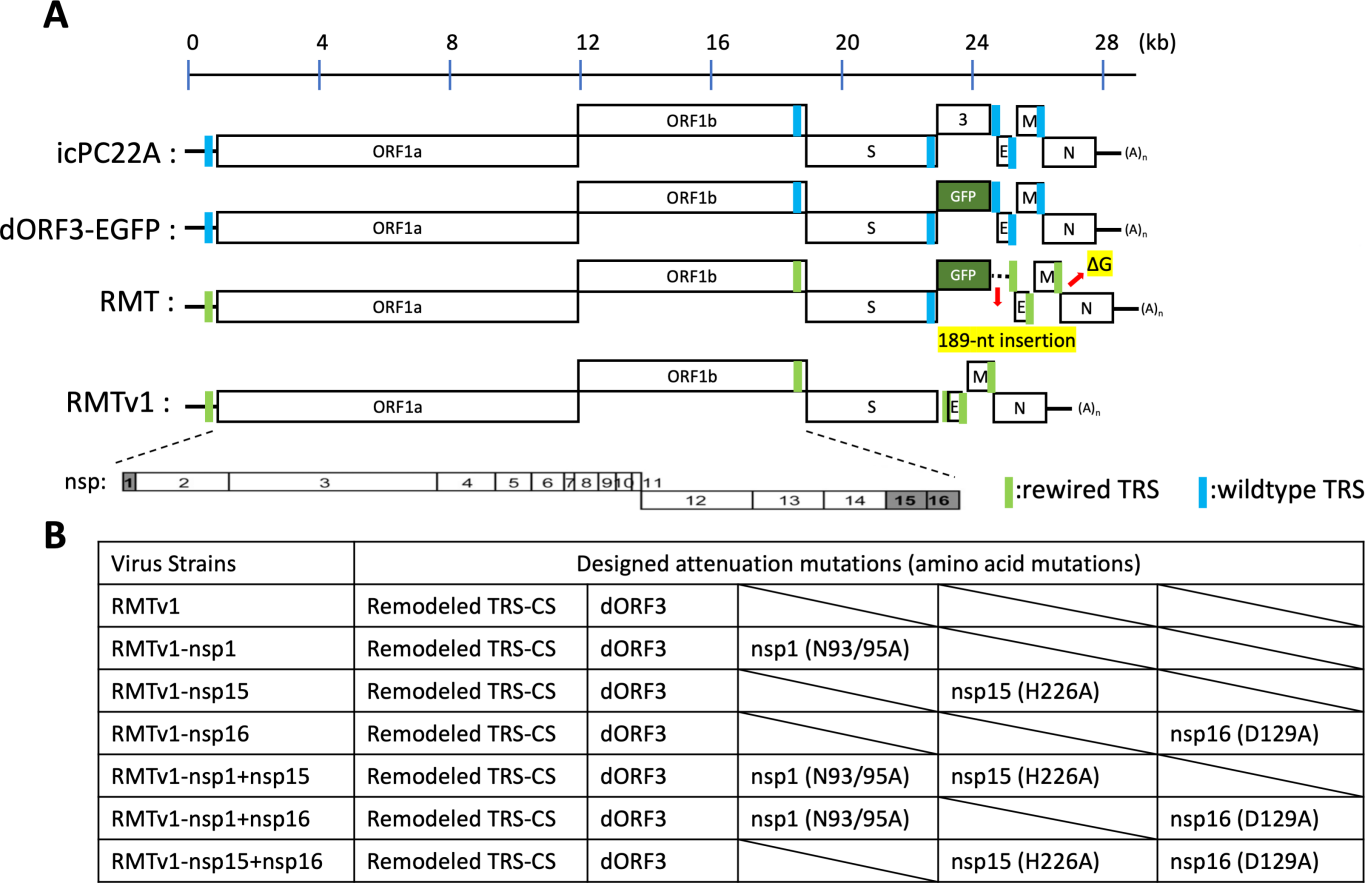
Schematic diagram of the genome organization of PEDV and positions of nucleotide changes in RMTv1 mutant viruses. (**A**) Schematic diagram of the genome organization of infectious clone-derived (ic) PC22A, and dORF3-EGFP, RMT and RMTv1, with the location of wild type (blue) and remodeled TRS-CSs (green). Two unexpected mutations (a 189-nt insertion in front of E gene TRS-CS and a ΔG in the TRS-CS of the N gene) in RMT mutant are pointed out with red arrows. Non-structural proteins 1, 15, and 16 are highlighted in gray. (**B**) The designed attenuation mutations of each mutant.

The 189-nt insertion in front of the E gene TRS-CS and one “G” deletion in the N gene TRS-CS in the RMT virus were fixed, and the EGFP reporter gene was removed to generate RMTv1 (Fig. 1A). The RMTv1 virus was rescued in Vero cells as passage 0 (P0). A single clone of the mutant was selected by plaque assay and passaged once in Vero cells to generate the P1 virus stock. The complete genome sequence of RMTv1-P1 was verified (Table 1)

**TABLE 1:** Genetic stability of RMTv1 and the viruses carrying one or two suppressed IFN antagonists

Virus strains	Clone no.^*a*^ and passage no.^*b*^	Mutations in 5′-UTR^*c*^	Non-synonymous mutations Viral protein (nt-AA)^*f*^^,*g*^	Synonymous mutations Viral protein (nt-AA)^*f*^^,*g*^
RMTv1	Clone 2 P1^*d*^			
	Clone 2 P10	g81t		
	Clone 4 P1		nsp2 (c1247t-L209F)	
RMTv1-nsp1	Clone 2 P1^*d*^		nsp10 (a12338t-N51Y)	
RMTv1-nsp15	Clone 2 P1^*d*^	c64t	nsp10 (a12338t-N51Y)	
RMTv1-nsp16	Clone 2 P1	c83t	nsp2 (t2539g-D639E) nsp3 (c2916a-T15K, a6546c-Q1225P)	nsp12 (a15159g-S370S)
	Clone 3 P1^*d*^	g81t		
RMTv1-nsp1 + nsp15	Clone 2 P1^*d*^	a76 deletion		nsp14 (c17427t-G88G)
	Clone 2 P10	a76 deletion; t98g		nsp14 (c17427t-G88G)
	Clone 3 P1	a76 deletion	Unknown^*e*^	Unknown^*e*^
RMTv1-nsp1 + nsp16	Clone 2 P1^*d*^	g73a		Spike (a20768c-A45A, c21005t-R124R)
	Clone 2 P10	g73a	Spike (t23532a-F967I)	Spike (a20768c-A45A, c21005t-R124R)
	Clone 5 P1	g73a	Unknown^*e*^	Unknown^*e*^
RMTv1-nsp15 + nsp16	Clone 1 P1^*d*^	a129c		
	Clone 1 P10	a129c; c64t	Spike (t23324a-F897L)	Spike (c23105t-L824L)
	Clone 3 P1	a76c		nsp16 (c20436t-S6S)

^*a*^ Five individual plaques per mutant were initially selected and propagated. The whole genomes of well-replicated ones with clear cytopathic effect were sequenced.

^*b*^ Clone with the fewest mutations (see footnote d) was passaged 10 times in Vero cells.

^*c*^ Single-nt substitution/deletion in 5′-UTR was detected near leader TRS core sequence (located at 67–72 nt).

^*d*^ The clone with the fewest mutations was used for the following experiments.

^*e*^ This clone cannot be better than the clone with the fewest mutations (see footnote d) and was not fully sequenced.

^*f*^ The nucleic acid mutations were numbered as their location in the whole PEDV genome.

^*g*^ The amino acid substitutions were numbered as their location in the specific protein.

Next, additional attenuation mutations located distantly were introduced in RMTv1. We chose PEDV nsp1 N93/95A, nsp15 H226A, and nsp16 D129A mutations that had been reported to suppress these IFN antagonists (51–54). We generated six mutants (RMTv1-nsp1, RMTv1-nsp15, RMTv1-nsp16, RMTv1-nsp1 + nsp15, RMTv1-nsp1 + nsp16, and RMTv1-nsp15 + nsp16) that contain single or double mutations among nsp1, nsp15, or nsp16 based on the RMTv1 backbone (Fig. 1B). Five plaques of each mutant were picked up by plaque assay. The whole genomes of well-replicated ones with clear cytopathic effects (CPEs) were sequenced. These IFN antagonist mutants, except for RMTv1-nsp1, had unexpected single-nucleotide substitution/deletion in the 5′-UTR near the leader TRS-CS (located at nt 67–72) (Table 1). We selected the clone with the fewest mutations of each virus for the following studies. We failed to rescue the designed RMTv1-nsp1 + nsp15 + nsp16 virus.

### Rewired TRS-CS and the targeted IFN antagonist mutations were stable after serial passage *in vitro*

To evaluate the genetic stability of the mutant viruses, the RMTv1 and all three mutants with mutations in two of the three IFN antagonists (nsp1, nsp15, and nsp16) were serially passaged in Vero cells nine times to the P10. Whole genomic sequences of the P10 viruses were determined (Table 1). After 10 passages of all mutants, there were no reversion mutations observed in the rewired TRS-CS and the targeted IFN antagonists. However, RMTv1 P10, RMTv1-nsp1 + nsp15 P10 and RMTv1-nsp15 + nsp16 P10 evolved one additional substitution g81t, t98g, and c64t, respectively, near the leader TRS-CS, suggesting that these 5′-UTR mutations near the leader TRS-CS likely provide a selective advantage for viral survival. Additionally, non-synonymous mutations were also detected in the spike protein of RMTv1-nsp1 + nsp16 P10 (F967I) and RMTv1-nsp15 + nsp16 P10 (F897L).

### Replication of RMTv1 and the six mutants with single or double IFN antagonist mutations in Vero cells and LLC-PK1 cells

**Fig 2:**
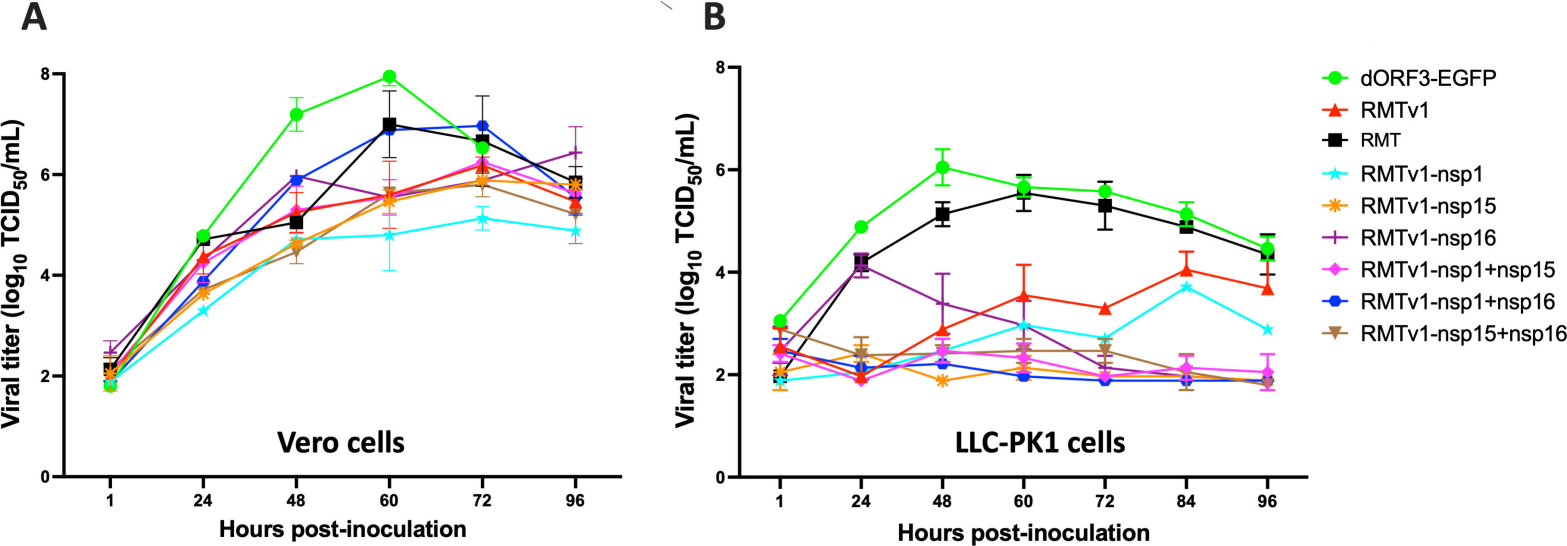
Multistep growth kinetics of PEDV mutants in Vero cells (**A**) and LLC-PK1 cells (**B**). Cells were infected with each virus at an MOI of 0.01. The titer of infectious virus was determined in Vero cells using a TCID_50_ assay, and the results were shown in mean ± standard deviations.

The multistep growth kinetics of the PEDV mutants were performed with a multiplicity of infection (MOI) = 0.01. In Vero cells, RMTv1 reached the peak infectious titer at 72 h post-inoculation (hpi), which was slower than RMT (60 hpi) and dORF3-EGFP (60 hpi). In addition, the RMTv1 mutant replicated to a lower peak infectious titer (6.19 ± 0.16 50% tissue culture infective dose [TCID_50_]/mL) than RMT (7.00 ± 0.66 TCID_50_/mL) and dORF3-EGFP (7.95 ± 0.18 TCID_50_/mL) (Fig. 2A). Similarly, in LLC-PK1 cells, RMTv1 reached the peak titer slower (84 hpi vs 60 hpi and 48 hpi), and its peak infectious titer (4.05 ± 0.35 TCID_50_/mL) was lower than those of RMT (5.55 ± 0.35 TCID_50_/mL) and dORF3-EGFP (6.05 ± 0.35 TCID_50_/mL) (Fig. 2B).

**Fig 3:**
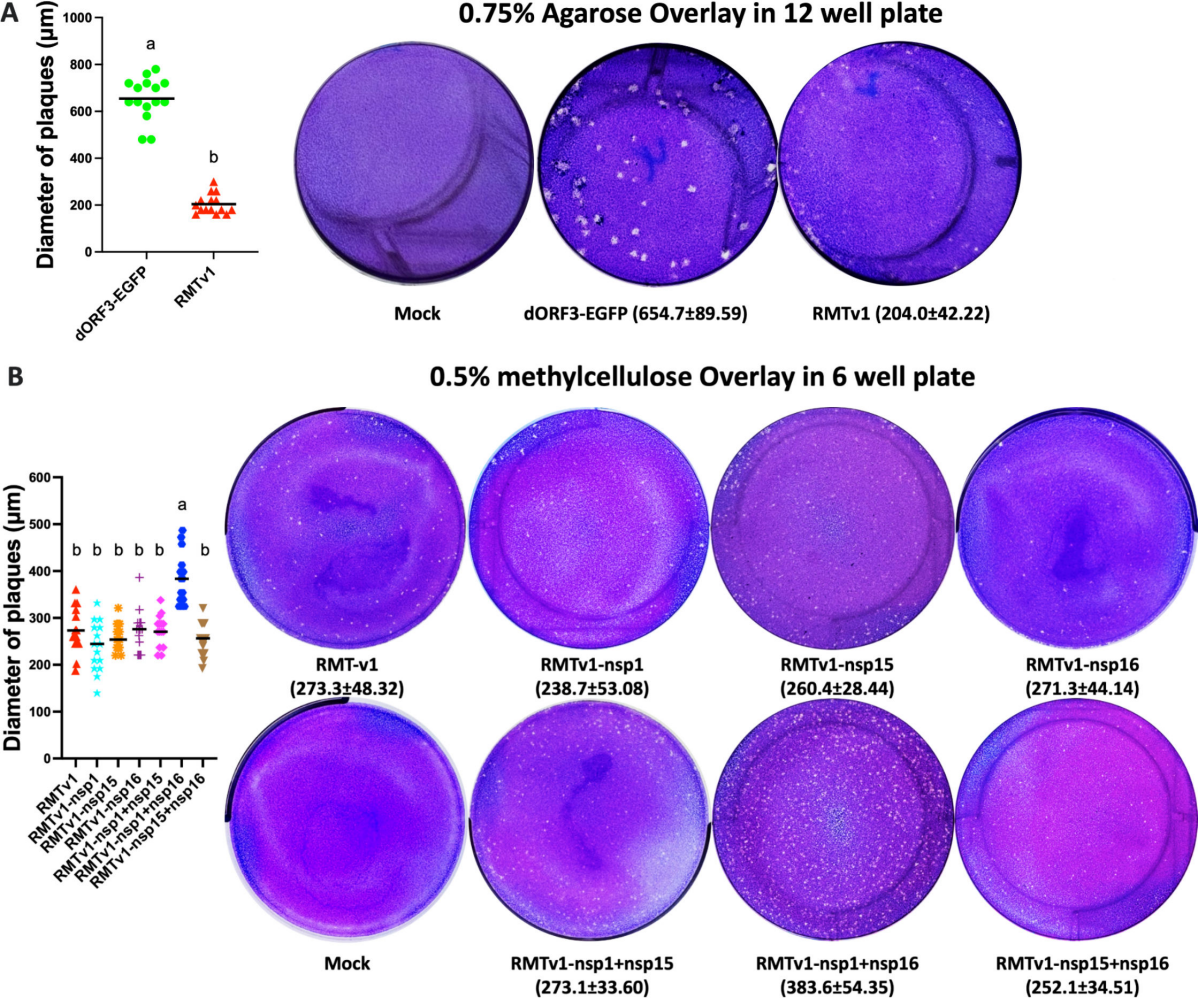
Plaques of PEDV mutants in Vero cells. Final concentration (0.75%) of agarose overlay (**A**), and final concentration (0.5%) of methylcellulose overlay (**B**) were used. The cells were fixed and stained at 4 days post-inoculation. The diameters of 15 plaques were measured and plotted. The mean is shown as a line. Results are presented in mean ± standard deviation. Groups with significant differences (*P* < 0.05) are indicated by different letters.

Five of the six IFN antagonist mutants showed comparable replication kinetics with RMTv1 in Vero cells (Fig. 2A), except for one outlier RMTv1-nsp1 + nsp16 that replicated to a peak titer similar to that of RMT, likely due to the g73a mutation right next to the leader TRS-CS in 5′-UTR (Table 1). These results are consistent with the concept that interferon antagonists are not required for virus replication in interferon-deficient cells. The plaque sizes of PEDV mutants correlated with their peak titers in Vero cells. RMTv1 formed smaller plaques than dORF3-EGFP (Fig. 3A). The outlier RMTv1-nsp1 + nsp16 formed bigger plaques than RMTv1, while the other five mutants and RMTv1 generated similar sizes of plaques in Vero cells (Fig. 3B).

In contrast to the results in Vero cells, we found that the levels of replication of IFN antagonist mutants were significantly reduced in interferon-sufficient LLC-PK1 cells compared with RMTv1, especially the three mutants with double nsp mutations (Fig. 2B). At 24 hpi, RMTv1-nsp16 replicated more efficiently than the other nsp mutants in LLC-PK1 cells (Table 1). RMTv1-nsp1 mutant replicated to a higher titer at 84 hpi than the other five mutants.

### The RMTv1 and double IFN antagonist mutants induced stronger IFN responses and were more sensitive to IFN-β pre-treatment than wild-type PEDV *in vitro*

**Fig 4:**
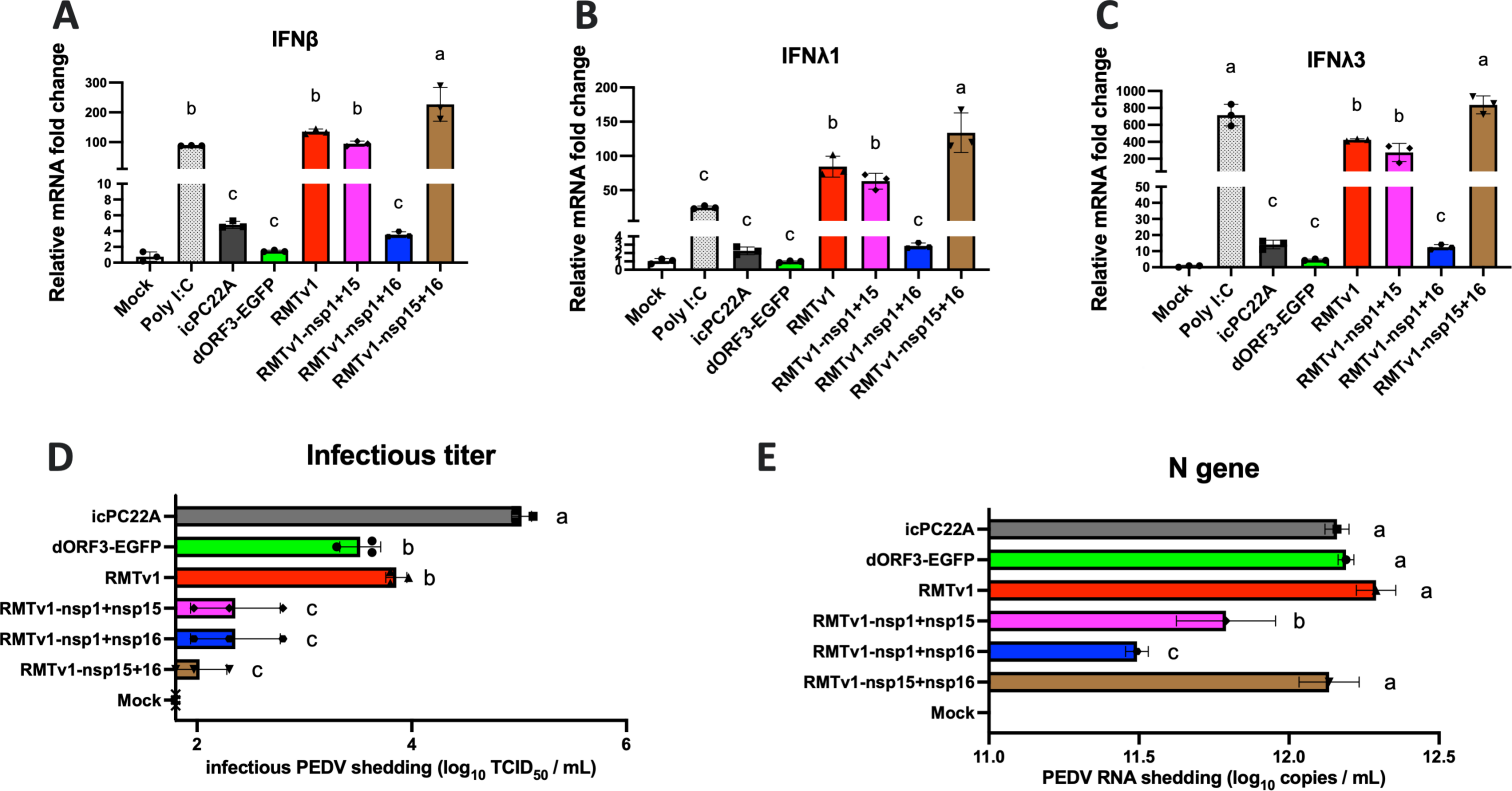
Induction of type I and type III IFN mRNAs by the PEDV mutants in LLC-PK1 cells. The cells were inoculated with each virus at an MOI of 1 or medium (mock) or stimulated with poly I:C as a positive control. At 12 hpi, the supernatants were collected for testing viral infectious titers, and cells were lysed for measuring IFN mRNA levels, including IFN-β (**A**), IFN-λ1 (**B**), and IFN-λ3 (**C**). The viral infectious titers in TCID_50_ (**D**) and PEDV total N gene RNA levels are shown (**E**). Different letters denote statistically significant differences (*P* < 0.05).

In LLC-PK1 cells, RMTv1 induced significantly higher IFN-β, IFN-λ1, and IFN-λ3 levels than the parental dORF3-EGFP and the infectious clone-derived wild-type PEDV strain icPC22A, likely due to the remodeled TRS-CS (Fig. 4A through C). The introduction of additional suppressive mutations in the IFN antagonists (nsp1, nsp15, and nsp16) was expected to further enhance IFN responses. Indeed, RMTv1-nsp15 + nsp16 stimulated significantly higher IFN responses compared with RMTv1 as expected. However, RMTv1-nsp1 + nsp15 induced similar IFN levels to RMTv1. RMTv1-nsp1 + nsp16 induced comparable IFN levels with dORF3-EGFP and icPC22A, which were significantly lower than RMTv1, despite carrying additional suppressive mutations in two IFN antagonists. A potential explanation for this discrepancy is that unexpected single nt substitutions or deletions (Table 1) closer to the leader TRS-CS (nt 67–72) exert a stronger compensatory effect on IFN induction.

We also evaluated viral replication by measuring infectious viral titers in the supernatants (Fig. 4D) and quantifying PEDV total N gene RNA levels in cell lysates (Fig. 4E). The infectious titers showed a clear trend: icPC22A exhibited the highest titer, followed by dORF3-EGFP and RMTv1, which had comparable titers, while all three double IFN antagonist mutants (RMTv1-nsp1 + nsp15, RMTv1-nsp1 + nsp16, and RMTv1-nsp15 + nsp16) displayed significantly reduced titers. This pattern reflects the progressive attenuation effect associated with the accumulation of mutations. As for the viral N gene RNA level, RMTv1-nsp1 + nsp15 exhibited significantly lower viral RNA levels than RMTv1, consistent with stronger attenuation due to the suppression of IFN antagonists. Interestingly, RMTv1-nsp1 + nsp16 had even lower viral RNA levels than RMTv1-nsp1 + nsp15 despite their infectious titers being similar, suggesting that the g73a mutation in the 5′-UTR may enhance propagation efficiency, allowing the virus to produce infectious particles more effectively despite reduced overall RNA levels. These results are consistent with the observed lower IFN responses, as a higher ratio of viral RNA shedding titer to infectious titer could correlate with enhanced innate immune activation (51). Unlike RMTv1-nsp1 + nsp15 and RMTv1-nsp1 + nsp16, RMTv1-nsp15 + nsp16 displayed similarly high levels of N gene to icPC22A, dORF3-EGFP, and RMTv1 but had the lowest infectious viral titers among all these mutants, indicating more defective RNA and inefficient propagation of infectious viral particles. This reduced propagation efficiency likely resulted from the combination of the suppressive mutations of IFN antagonists and the weak compensatory effect of the a129c mutation located farthest from the leader TRS-CS. This inefficiency in propagation correlates with the strongest IFN induction among the viruses. Moreover, the robust IFN responses triggered by RMTv1-nsp15 + 16 infection may in turn restrict viral propagation.

**Fig 5:**
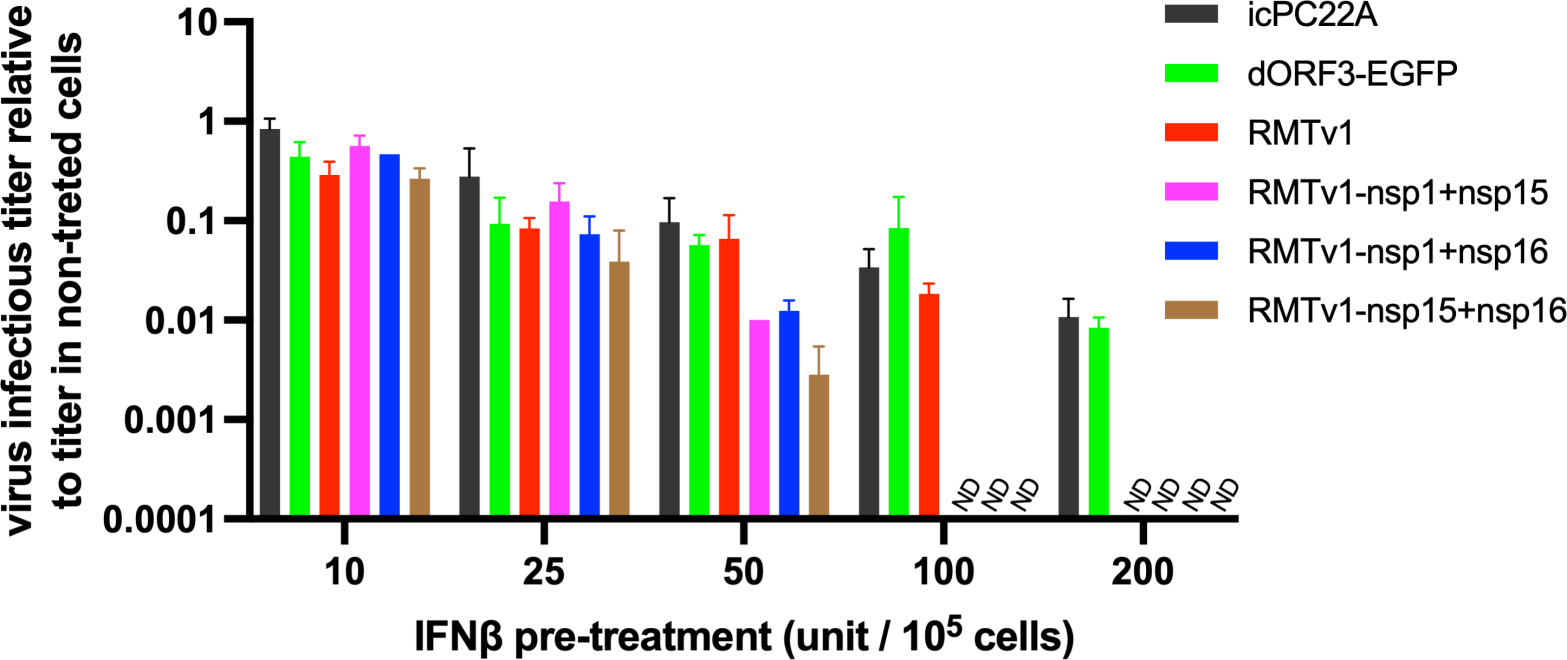
Sensitivity of the PEDV mutants to IFN-β pre-treatment in Vero Cells. Cells were pre-treated with different concentrations of IFN-β for 18 h and inoculated with individual viruses at an MOI of 0.01. Values indicate the ratio of the viral infectious titers of the treated cells relative to those of the non-treated cells at 24 hpi. ND, non-detectable.

Although Vero cells do not produce IFN, they retain IFN receptor signaling pathways. Thus, they are a standard model to assess IFN-β sensitivity in PEDV research (55). We also compared the replication of these mutants in Vero cells that were pre-treated with different concentrations of IFN-β (Fig. 5). IFN-β pre-treatment inhibited the replication of all the viruses in a dose-dependent manner. We found that RMTv1 variants with double nsp mutations were more sensitive to IFNβ treatment than RMTv1, followed by icPC22A and dORF3-EGFP. This could explain why the RMTv1-nsp1 + nsp15 and RMTv1-nsp1 + nsp16 replicated to significantly lower infectious titers in LLC-PK1 cells despite inducing similar and lower IFN levels to RMTv1, respectively (Fig. 2 and 4).

### RMTv1 and RMTv1-nsp1 + nsp15 mutants were attenuated in neonatal Gn piglets

We selected RMTv1-nsp1 + nsp15 for *in vivo* study because it carries multiple attenuation mutations distributed distantly along the genome, reducing the likelihood of reversion to virulence through a single mutation. Among the double mutants, RMTv1-nsp1 + nsp15 showed no amino acid mutations after 10 passages in Vero cells, whereas RMTv1-nsp1 + nsp16 and RMTv1-nsp15 + nsp16 accumulated mutations in spike protein (Table 2). In addition, RMTv1-nsp1 + nsp15 induced higher IFN levels than icPC22A and similar IFN levels to RMTv1 (Fig. 4).

**TABLE 2:** Summary of clinical signs and fecal PEDV RNA shedding of Gn piglets from 1 to 10 days post-inoculation

Group	No. of pigs^*a*^	Fatality rate (% [no./total])	Severe diarrhea rate (% [no./total])^*b*^	Onset of diarrhea (dpi)^*b*^	Peak mean RNA shedding titer (log_10_ N gene copies/mL)
icPC22A	4	100 (4/4)	100 (4/4)	1.00 ± 0.00 A^*c*^	11.93 ± 0.43 (1 dpi) A
RMTv1	4	75 (3/4)	100 (4/4)	2.00 ± 0.00 B	9.60 ± 0.39 (2 dpi) B
RMTv1-nsp1 + nsp15	5	0 (0/4)	0 (0/4)	2.50 ± 0.58 B	8.28 ± 0.48 (6 dpi) C
Mock	4	0 (0/4)	0 (0/4)	NA^*d*^	NA^*d*^

^*a*^ The pigs from the RMTv1-nsp1 + nsp15 group that were euthanized at 4 dpi for histopathological examination were excluded from the calculation of mortality and diarrhea rates, onset of diarrhea, and peak mean RNA shedding titers.

^*b*^ Fecal consistency scores of 2 and 3 were considered moderate diarrhea and severe diarrhea, respectively.

^*c*^ Different letters denote significant difference between groups (*P* < 0.05).

^*d*^ NA, not available.

**Fig 6:**
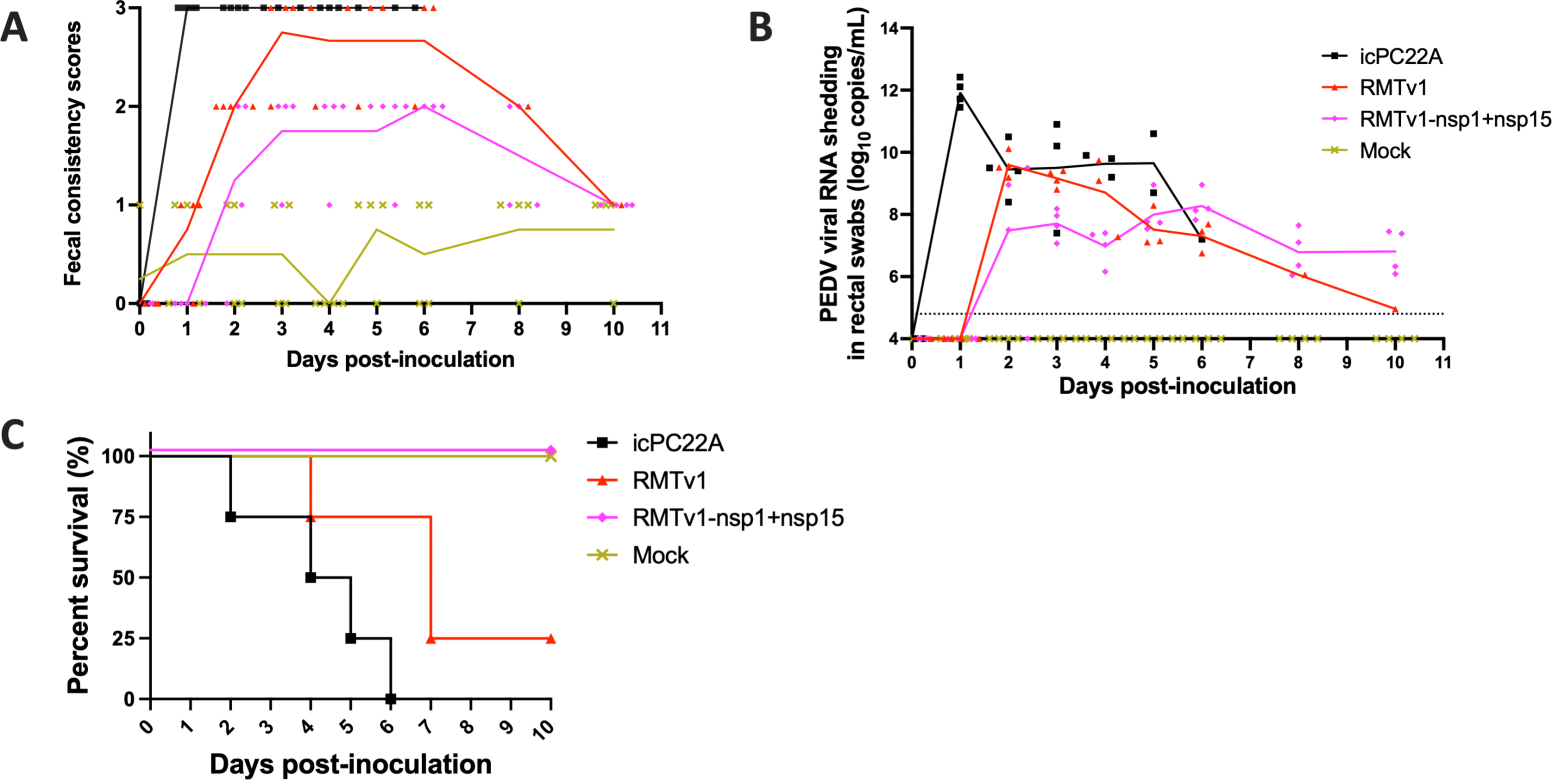
Evaluation of the replication and pathogenesis of the infectious clone-derived viruses in 3-day-old Gn piglets. Each line represents the mean value of each group, and the values of individual Gn piglets are also shown: (**A**) fecal consistency scores (0, solid; 1, pasty; 2, semiliquid; and 3, liquid). A score of ≥2 was considered diarrhea. (**B**) fecal PEDV RNA shedding profile. The dashed line at 4.80 log_10_ copies/mL indicates the detection limit and (**C**) survival curves of groups inoculated with PEDV mutants.

The pathogenicity of the PEDV mutants was evaluated in four groups of 3-day-old Gn piglets (Table 2; Fig. 6). Piglets infected with the virulent icPC22A strain exhibited severe clinical signs, including a 100% fatality rate (four out of four) and persistent severe diarrhea beginning at 1 dpi, with the highest peak viral RNA shedding titer of 11.93 ± 0.43 log_10_ copies/mL at 1 dpi. RMTv1-infected piglets displayed similar but slightly attenuated pathogenicity, with a delayed onset of diarrhea at 2 dpi and a lower peak viral RNA shedding titer of 9.60 ± 0.39 log_10_ copies/mL. However, RMTv1 still caused severe diarrhea in all infected piglets (four out of four), and 75% (three out of four) of the pigs died. In contrast, piglets inoculated with RMTv1-nsp1 + nsp15 mutant exhibited no severe diarrhea and no mortality, but the piglets still had moderate diarrhea with a delayed onset at 2.50 ± 0.58 dpi. The peak viral RNA shedding titer in this group was lower (8.28 ± 0.48 log_10_ copies/mL) and delayed at 6 dpi compared with RMTv1, indicating attenuated viral replication. Mock piglets remained clinically normal, with no signs of diarrhea or viral shedding. These findings confirm that while RMTv1 is slightly attenuated compared with icPC22A, RMTv1-nsp1 + nsp15 demonstrates a markedly attenuated phenotype.

### Histopathological examination

**Fig 7:**
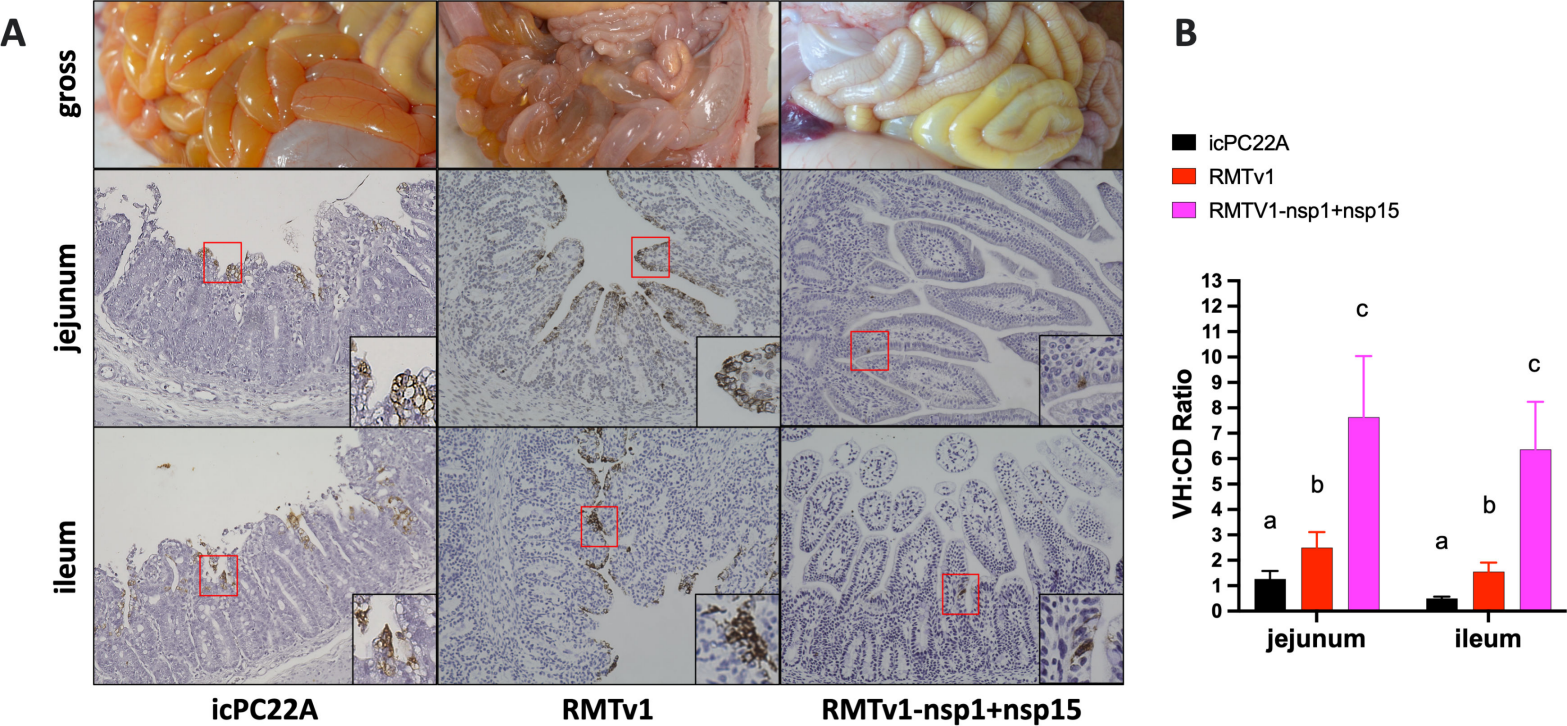
Histopathological examination of the small intestines of Gn piglets euthanized at 4 dpi. (**A**) The gross image of pig intestines and the immunohistochemical staining of PEDV N proteins (brown signals) in the jejunum and ileum sections of the piglets (magnification, ×200). (**B**) Villous height-to-crypt depth (VH:CD) ratios of piglets. Twenty villi of each intestinal section were measured. Groups with significant differences (*P* < 0.05) were indicated with different letters. The error bars indicate standard deviations.

The RMTv1-infected piglets exhibited moderate villous atrophy in the jejunum and ileum, and the villous height-to-crypt depth (VH:CD) ratios were slightly higher than those in icPC22A-infected piglets, indicating partial attenuation (Fig. 7). As expected, RMTv1-nsp1 + 15-infected piglets showed markedly less severe villous atrophy and significantly higher VH:CD ratios than the pigs in RMTv1 and icPC22A groups, suggesting significant attenuation. Immunohistochemical staining for PEDV N proteins showed reduced viral antigen presence in the intestines of RMTv1-nsp1 + 15-infected piglets compared with those infected with RMTv1 and icPC22A, correlating with the attenuated clinical phenotype (Fig. 7A).

### RMTv1-nsp1+15 mutants induced protection against virulent icPC22A challenge in pigs

**Fig 8:**
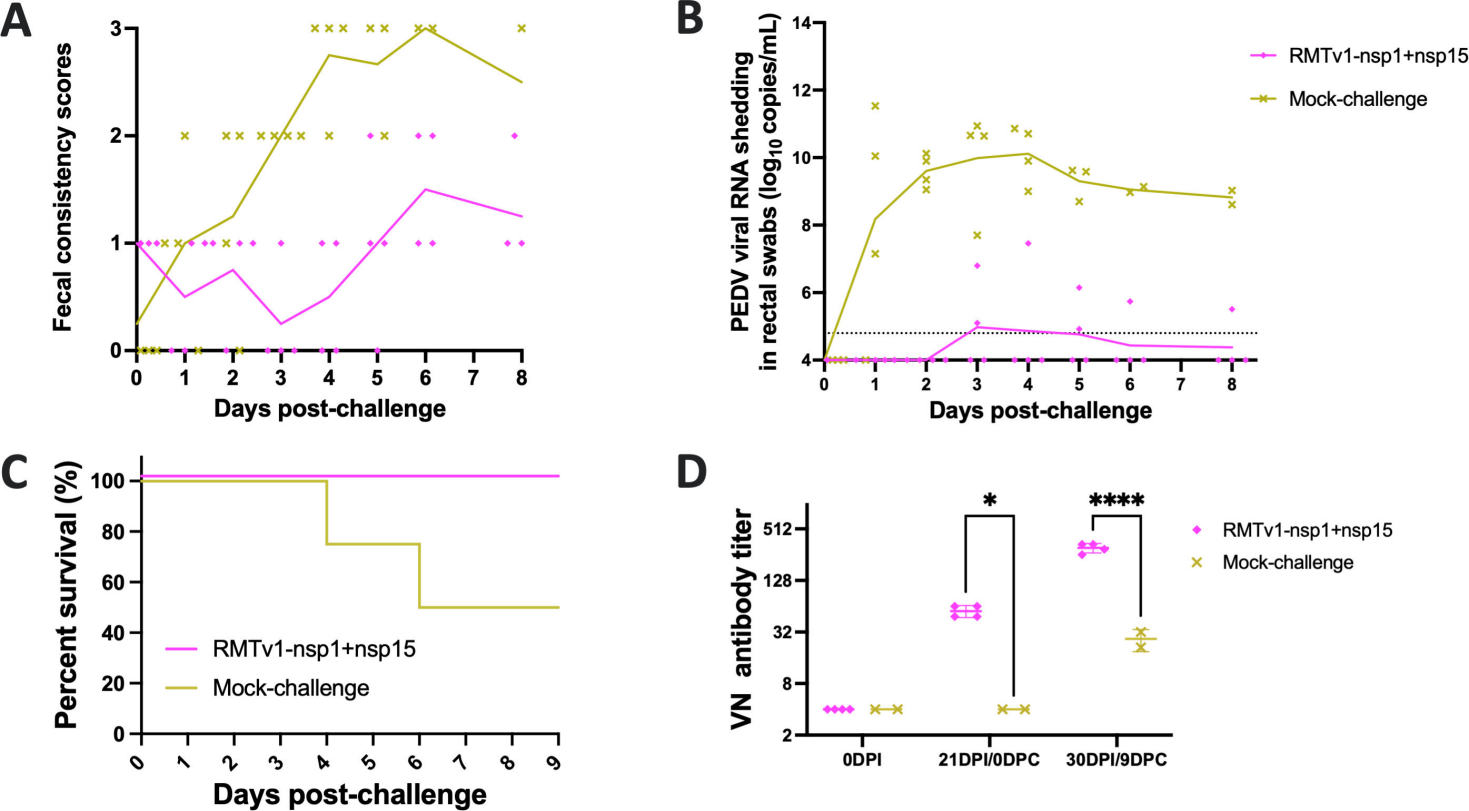
Induction of protection by RMTv1-nsp1 + nsp15 mutant in Gn pigs against icPC22A challenge. (**A**) Fecal consistency scores of pigs post-challenge. Fecal consistency was scored as follows: 0, solid; 1, pasty; 2, semiliquid; and 3, liquid. Scores of 2 and 3 were considered diarrhea and severe diarrhea, respectively. Each dot represents the score of an individual pig; each line indicates the mean score of a group. (**B**) Fecal PEDV RNA (N gene) shedding titers post-challenge. Each symbol represents the titer of an individual piglet; each line indicates the mean value of a group. The dashed line at 4.80 log_10_ copies/mL indicates the detection limit. (**C**) Survival curves of pigs by 9 dpc. (**D**) Viral neutralizing (VN) antibody titers in the sera collected at different time points. Each symbol represents the titer of an individual piglet. Data are shown as mean ± standard deviation (*, *P* < 0.05; ****, *P* < 0.001)

To evaluate the immunogenicity and protective efficacy of the attenuated RMTv1-nsp1 + nsp15 mutant, piglets were challenged with a high dose (10^6^ TCID_50_/pig) of the virulent icPC22A strain at 21 dpi. Following the challenge, mock-challenge piglets exhibited severe diarrhea lasting for 2.0 ± 1.41 days, and a 50% (two out of four) fatality rate (Table 3; Fig. 8). In contrast, RMTv1-nsp1 + nsp15-infected and challenged piglets had no severe diarrhea and no mortality. Although 50% of the pigs still had mild diarrhea (two out of four), the duration of diarrhea was significantly shorter in this group (2.00 ± 1.41 days) than that (4.50 ± 1.00 days) in the mock-challenge group. Viral RNA shedding post-challenge was dramatically reduced in the piglets of the RMTv1-nsp1 + nsp15 group, with a peak mean titer (4.98 ± 1.32 log_10_ N gene copies/mL) just above the detection limit at 4 days post-challenge (dpc), which was significantly lower than the 9.99 ± 1.53 log_10_ copies/mL detected in the pigs in the mock-challenge group at 3 dpc. Furthermore, the RMTv1-nsp1 + nsp15-infected pigs developed robust viral neutralizing (VN) antibody responses at challenge day (21 dpi/0 dpc), and the titer was boosted post-challenge at 30 dpi/9 dpc (Fig. 8D). On the other hand, the pigs in the mock-challenge group did not have detectable VN antibodies at challenge day and had low levels of VN antibody titers at 9 dpc. These findings indicate that RMTv1-nsp1 + nsp15 retained good immunogenicity and elicited strong VN antibody responses and provided substantial protection against lethal icPC22A challenge.

**TABLE 3:** Pig clinical signs and fecal PEDV RNA shedding levels post-challenge with the highly virulent icPC22A strain (1–9 days)

Group	No. of pigs	Fatality rate (%[no./total])	Diarrhea rate (% [no./total])^*a*^	Severe diarrhea rate (% [no./total])^*a*^	Duration of diarrhea, FC^*c*^ ≥2 (days)^*a*^	Duration of severe diarrhea, FC = 3 (days)^*a*^	Peak mean RNA shedding titer (log_10_ N gene copies/mL)
RMTv1-nsp1 + nsp15	4	0 (0/4)	50 (2/4) A^*b*^	0 (0/4) A	2.00 ± 1.41 A	0.00 ± 0.00 A	4.98 ± 1.32 (4 dpc) A
Mock challenge	4	50 (2/4)	100 (4/4) B	100 (4/4) B	4.50 ± 1.00 B	2.00 ± 1.41 B	9.99 ± 1.53 (3 dpc) B

^*a*^ FC scores of 2 and 3 were considered moderate diarrhea and severe diarrhea, respectively.

^*b*^ Different letters denote significant difference between groups (*P* < 0.05).

^*c*^ FC, fecal consistency.

## DISCUSSION

The development of safe and effective LAVs for PEDV is urgently needed due to the severe economic impact PEDV outbreaks have on the global swine industry. However, the accumulation of mutations and/or recombination of a LAV with field strains can lead to vaccine failure. Our laboratory’s long-term goal is to resolve these problems of PEDV LAVs using reverse genetics technology. First, we generated a recombination-resistant PEDV RMT mutant (43). The RMT had extensively disrupted transcription circuits, including a dramatically increased sgmRNA-E level, compared with the parental virus dORF3-EGFP (43). It is unclear whether such different transcription profiles were caused by the remodeled TRS-CSs and/or the two unexpected mutations, which need further characterization in the future. In this study, we further revised RMT to generate RMTv1. Next, we used RMTv1 as the vaccine platform to develop LAV candidates for PEDV.

Interestingly, we observed that RMTv1 replicated less efficiently than dORF3-EGFP (Fig. 2) and induced stronger IFN responses (Fig. 4). These findings suggest that remodeling the TRS-CS to create a recombination-resistant LAV platform may disturb viral discontinuous transcription and increase the production of defective viral RNA as pathogen-associated molecular patterns (PAMPs), potentially inducing stronger innate immune responses. This observation aligns with the known mechanism of coronavirus discontinuous transcription. The stability of the complementary interactions between leader and body TRS-CS regions is a key determinant of the efficiency and frequency of sgmRNA transcription (45, 47). Previous studies have demonstrated that altering conserved nucleotides within the CS of TRS-L or TRS-B can expose novel complementary sites within the viral genome, leading to the production of aberrant small sgRNAs (56, 57). These aberrant sgRNAs disrupt the transcription of essential sgmRNAs necessary for viral protein synthesis, thereby impairing viral replication or rendering the virus non-viable. Therefore, we searched the PEDV genome for sequences totally identical to the remodeled TRS-CS and introduced silent mutations to disrupt them (43). Nevertheless, the remodeled TRS-CS may inadvertently affect the accuracy of viral discontinuous transcription because the overall pairing affinity between TRS-B and TRS-L is determined not only by TRS-CS but also the surrounding sequences, whose function is largely unknown. Also, how many nucleotide mismatches this TRS-B and TRS-L pairing process can tolerate remains unknown. Although we removed all the sequences identical to the remodeled TRS-CS along the PEDV genome, the presence of similar sequences with mismatches remains unavoidable. Eventually, the remodeled TRS-CS could reduce replication efficiency and increase PAMP generation. The underlying mechanisms driving these effects merit further investigation to optimize the balance between recombination resistance, viral attenuation, replication competence, and immunogenicity in future vaccine development.

To further attenuate the virus for LAV development, we introduced mutations in nsp1 (51), nsp15 (52), and nsp16 (53) that are key interferon antagonists contributing to viral replication and virulence. Briefly, coronavirus nsp1 suppresses host gene expression by degrading host mRNA; nsp15 exhibits endoribonuclease activity that specifically recognizes the uridylates and excises both ssRNAs and dsRNAs; and nsp16 catalyzes the 2′-O methylation for viral RNA capping. PEDV mutants carrying suppressive mutations in these interferon antagonists were reported to generate an earlier and more robust interferon response in cell culture than the wild-type virus and were attenuated in piglets. An icPEDV-mut4 mutant carrying a combination of attenuated versions of nsp1 (F44A), nsp15 (H226/241A), and nsp16 (D129A) was reported previously (54). This mutant elicited robust interferon responses and exhibited severely impaired replication in LLC-PK1 cells. In 1-week-old piglets, icPEDV-mut4 was attenuated and induced IgG and VN antibodies. However, no challenge study was conducted. In our study, the RMTv1 backbone already included remodeled TRS-CS and ORF3 deletion. In addition, our nsp1 mutant contained two amino acid substitutions at different locations compared to icPEDV-mut4. We were unable to introduce suppressive mutations in nsp1, nsp15, and nsp16 together in RMTv1, likely due to the excessive attenuation burden.

Also, the presence of multiple attenuation mutations at distinct genomic locations, in addition to the remodeled TRS-CS, reduces the likelihood of reversion to virulence, as a single mutation would be insufficient to restore full pathogenicity. However, introducing multiple mutations may lead to excessive attenuation and make the strain too weak to replicate well and induce sufficient protective immunity, whereas insufficient attenuation could compromise vaccine safety. Therefore, to achieve the balance between attenuation and immunogenicity, an optimal LAV should incorporate multiple attenuating mutations that are individually mild but collectively achieve the desired level of attenuation. This approach ensures that no single mutation drastically weakens the virus and maintains its ability to stimulate robust immune responses. Our *in vitro* studies showed that RMTv1-nsp1 + nsp15 (carrying rewired TRS-CS, deleted ORF3, and the suppressive mutations of nsp1 and nsp15) exhibited significantly reduced replication efficiency and induced stronger IFN responses compared with the dORF3-EGFP and icPC22A. Subsequent *in vivo* studies further confirmed the attenuation and protective efficacy of this vaccine candidate. Our results highlight the potential of RMTv1 as a recombination-resistance LAV platform and RMTv1-nsp1 + nsp15 as a promising LAV candidate for PEDV. However, because sows and gilts are less susceptible to PEDV than suckling piglets (58, 59), further studies are needed to determine whether this vaccine candidate can replicate efficiently in adult pigs and induce sufficient lactogenic immunity to protect their offspring.

**Fig 9:**
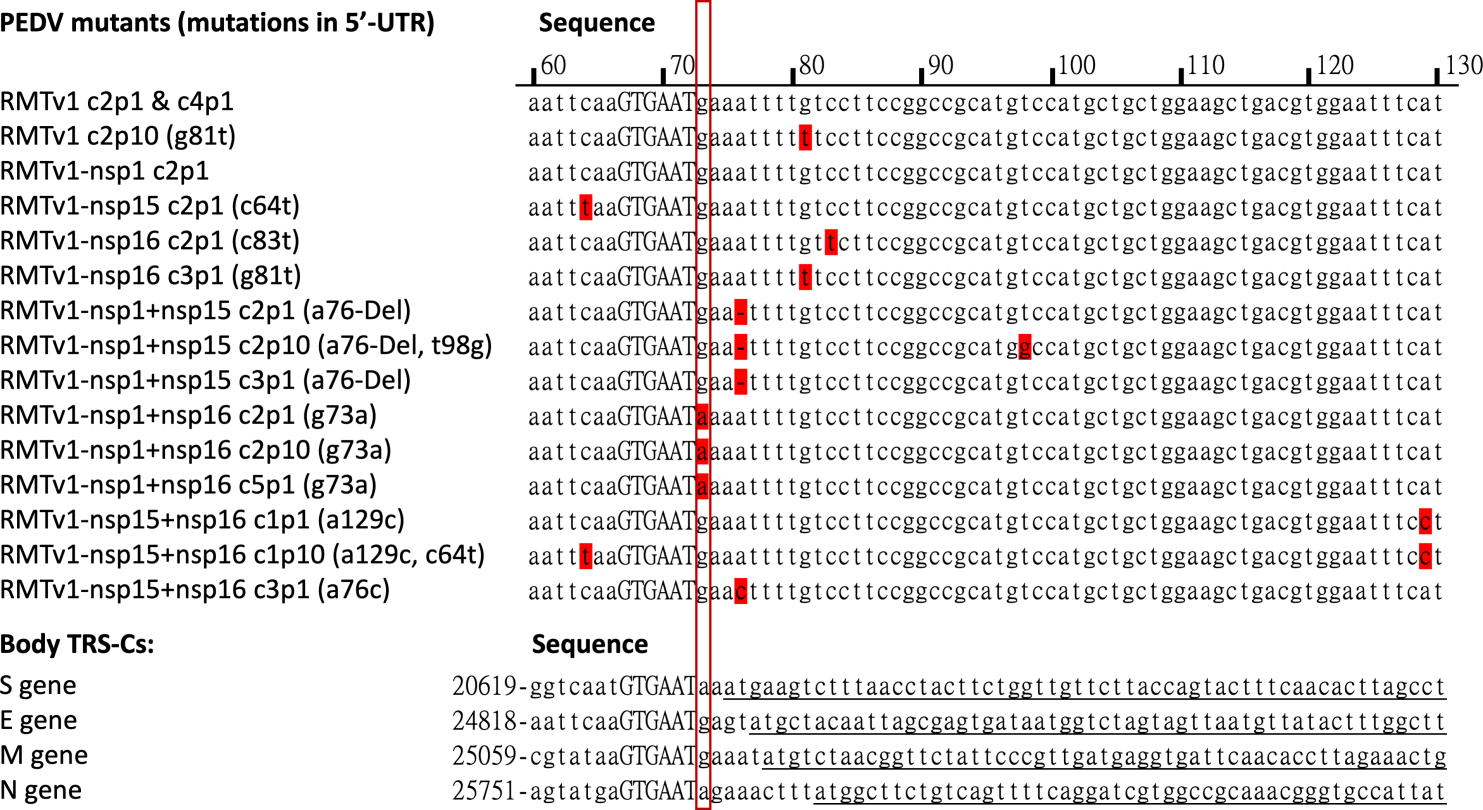
Rewired leader and body TRS-CS and flanking sequences in PEDV mutants. The rewired TRS-CS sequence “GTGAAT” is shown in upper case, and the mutations that evolved during virus rescue and passaging are highlighted in red. Nucleotide positions in the PEDV genome were indicated by numbers. Protein-encoding genes were underlined. The nucleotides immediately downstream of TRS-CS are boxed in red rectangles.

Interestingly, in addition to the targeted mutations, we observed that RMTv1 with suppressive nsp mutations frequently evolved single-nucleotide mutations (substitutions or deletions) in the 5′-UTR near the leader TRS-CS as early as P0. After 10 passages in Vero cells, similar mutations were also detected in RMTv1 P10, suggesting that the 5′-UTR mutations likely provide a selective advantage for viral survival; otherwise, they would not have been maintained or competed out during virus rescue, plaque purification, and serial passaging. Notably, these mutations appear to enhance viral replication and IFN induction (Fig. S1), possibly compensating for the negative effects of remodeled TRS-CS. Comparison of RMTv1-c2p1 and RMTv1-c2p10 infections of LLC-PK1 cells further supported this (Fig. S1A). The only difference between these two viruses is the g81t substitution near leader TRS-CS. RMTv1-c2p10 infection induced significantly lower levels of IFN-β, IFN-λ1, and IFN-λ3 than RMTv1-c2p1. RMTv1-c2p10 replicated to significantly higher infectious viral titers than RMTv1-c2p1. In addition, the RMTv1-c2p10 formed bigger plaques than RMTv1-c2p1 in Vero cells, indicating better replication (Fig. S1B). These results indicated that these mutations in the 5′-UTR near leader TRS-CS can enhance viral replication and suppress the IFN induction, probably by reducing the production of defective viral RNA. The IFN induction level of RMTv1-c2p10 was still higher than the parental dORF3-EGFP, suggesting that the g81t mutation cannot fully compensate for the negative effects on virus replication caused by the remodeled TRS-CS. Furthermore, this compensatory effect seems to correlate with the proximity of the mutation to the TRS-CS: the closer the mutation, the stronger the observed compensation (Table 1; Fig. 9). For instance, RMTv1-nsp1 + nsp16 harbors a g73a substitution, located immediately adjacent to the leader TRS-CS (nucleotides 67–72). This mutation changes the sequence from 'GTGAATg' to 'GTGAATa,' extending its identity to the body TRS-CS of the S and N genes by one more nucleotide and resulting in an enhanced base pairing. This identity expansion may explain why RMTv1-nsp1 + nsp16 replicated more efficiently and formed significantly larger plaques than the other RMTv1-based mutants in Vero cells (Fig. 3). In LLC-PK1 cells, RMTv1-nsp1 + nsp16 produced fewer viral transcripts yet achieved infectious titers comparable with RMTv1-nsp1 + nsp15 (Fig. 4), also suggesting more efficient replication. The more efficient viral replication corresponds to the less defective RNA production. This may counterbalance the expected increase in IFN induction from nsp1 and nsp16 suppression mutations. In contrast, RMTv1-nsp1 + nsp15 carries an a76 deletion, which is positioned slightly further from the TRS-CS than the g73a mutation and did not make leader TRS-CS more identical with body TRS-CSs. Despite this, it may still exert a modest compensatory effect. Interestingly, even after suppressing nsp1 and nsp15, the IFN induction level of RMTv1-nsp1 + nsp15 remained comparable with RMTv1, rather than increasing as reported in previous studies (51, 52). This suggests that the a76 deletion might mitigate the expected enhancement of IFN responses. Meanwhile, RMTv1-nsp15 + nsp16 contains an a129c substitution, the most distal mutation from the leader TRS-CS in this study. This mutant showed a significant increase in IFN induction alongside reduced replication efficiency, as reported previously, consistent with a weaker compensatory effect compared with those mutations of RMTv1-nsp1 + nsp15 and RMTv1-nsp1 + nsp16. Although investigating the role of TRS-flanking mutations in viral transcription and replication was not the primary focus of this study, our observations highlight the potential significance of these mutations in TRS-CS flanking sequences on viral fitness and immune evasion, which need to be further studied in the future. For the purposes of this study, we confirmed that despite the a76 deletion in RMTv1-nsp1 + nsp15, the TRS-L region remains distinct from that of WT PEDV and does not confer strong compensatory effects. Therefore, RMTv1-nsp1 + nsp15 remains a viable candidate for our LAV development.

Although RMTv1 and RMTv1-nsp1 + nsp15 induced comparable IFN responses *in vitro*, their pathogenicity profiles in piglets were clearly distinct. This discrepancy likely reflects the multifactorial nature of viral attenuation. Both nsp1 and nsp15 are multifunctional viral proteins involved not only in antagonizing host IFN responses but also in maintaining viral fitness. Coronaviruses nsp1 facilitates viral replication by degrading host mRNA and suppressing host translation (60), while nsp15 functions as an endoribonuclease within the replication-transcription complex to process viral RNAs (61, 62). Therefore, suppression of their functions may attenuate the virus independently of IFN signaling. More importantly, RMTv1-nsp1 + nsp15 was significantly more sensitive to IFN-β pre-treatment than RMTv1 (Fig. 5), suggesting that even with similar levels of IFN induction, the host innate immune responses are more effective at inhibiting RMTv1-nsp1 + nsp15 than RMTv1. Additionally, the a76 deletion near the TRS-L region may contribute to altered transcriptional regulation, further enhancing the attenuation phenotype. Taken together, the observed attenuation of RMTv1-nsp1 + nsp15 likely resulted from the combined effects of TRS-CS remodeling, deletion of ORF3, nsp1 and nsp15 suppression mutations (both IFN dependent and IFN independent), and the accompanying 5′-UTR mutation.

The potential recombination of CoVs is always a concern when using LAVs. The implications of this study extend beyond PEDV to other coronaviruses and nidoviruses that rely on discontinuous transcription. The feasibility of TRS rewiring for the development of LAVs has been successfully demonstrated in several viruses, including severe acute respiratory syndrome coronavirus (SARS-CoV) (56, 63), SARS-CoV-2 (64), and porcine reproductive and respiratory syndrome virus (PRRSV) (65). The TRS-rewired viruses were attenuated and protected against lethal SARS-CoV challenge in mice (63). While a 3-nt rewired TRS-CS reverts via second-site mutation upon serial passaging, a 7-nt rewired TRS is more stable, suggesting that a more extensively rewired TRS might be essential for avoiding growth selection. A PRRSV mutant vaTRSall carrying rewired TRS circuits based on the genome of the PRRSV live vaccine strain vHuN4-F112 was generated (65). The virus with a genome carrying incompatible TRS-CSs cannot be rescued. Recombination analysis *in vitro* and *in vivo* showed that the incidence rates of mutation breakpoints and template-switching recombination in the vaTRSall and vZJqz21 co-infected groups were effectively reduced compared with the vHuN4-F112 and vZJqz21 co-infection group. This study proved our concept on the side. A highly attenuated SARS-CoV-2 carrying rewired TRS and deleted accessory proteins was also proposed as a LAV platform (64). However, the recombination rate of this platform has not been tested. Our RMTv1 backbone and its variants also highlight the feasibility of remodeling TRS-CS in creating recombination-resistant vaccines for PEDV that are both safe and capable of inducing strong protective immunity.

Future research should focus on further refining the balance between attenuation and immunogenicity, potentially through additional genetic modifications that enhance the safety and efficacy of the vaccine. Long-term studies on the durability of the immune response and the potential need for booster vaccinations will be essential to ensure sustained protection.

In conclusion, the development of RMTv1 and RMTv1-nsp1 + nsp15 represents a significant advancement in PEDV vaccine research. These vaccine candidates demonstrate that achieving high levels of attenuation without compromising immunogenicity is possible, providing a promising solution for controlling PEDV outbreaks and informing the development of vaccines for other coronaviruses.

## MATERIALS AND METHODS

### Cells and reagents

Vero cells (ATCC CCL-81) were cultured in Dulbecco’s modified Eagle’s medium (DMEM; Thermo Fisher Scientific, Waltham, MA, USA) in the presence of 5% fetal bovine serum (FBS; Hyclone, Logan, UT, USA) and antibiotic-antimycotic (100 U/mL penicillin, 100 µg/mL streptomycin, and 250 ng/mL amphotericin B; Thermo Fisher Scientific). LLC porcine kidney (LLC-PK1) cells (ATCC CL-101) were cultured in modified Eagle’s medium (MEM, Thermo Fisher Scientific) supplemented with 5% FBS, 1% non-essential amino acids (NEAAs, Thermo Fisher Scientific), 1% antibiotic-antimycotic, and 1% HEPES (Thermo Fisher Scientific). For PEDV propagation, Vero cells were maintained in DMEM supplemented with 0.3% tryptose phosphate broth (TPB; Sigma-Aldrich, St. Louis, MO, USA), antibiotics (100 U/mL penicillin and 100 µg/mL streptomyci; Thermo Fisher Scientific), and 10 µg/mL trypsin (Thermo Fisher Scientific) as described previously (66). LLC-PK1 cells were maintained in MEM supplemented with 1% antibiotic-antimycotic, 1% NEAA, 1% HEPES, and 10 µg/mL trypsin after the viral inoculation (43, 51).

### Generation of PEDV mutants

The RMTv1 and other PEDV mutants carrying one or two mutations targeting nsp1, nsp15, or nsp16 (Fig. 1) were subjected to recovery based on the full-length cDNA clone of PEDV strain PC22A as described previously (53). Briefly, the point mutations were introduced into the plasmid pUC19 carrying the gene fragment of icPC22A (GenBank no. KY499262) via NEB Q5 Site-Directed Mutagenesis Kit (NEB, Ipswich, MA). The large piece of gene was inserted or deleted using the NEBuilder HiFi DNA Assembly Cloning Kit (NEB). Then, the sequence of plasmids was confirmed by the Whole Plasmid Sequencing service at Plasmidsaurus (Eugene, OR, USA) using Oxford Nanopore Technology. After the plasmids were digested by restriction enzymes, the appropriately sized cDNA inserts were purified using the QIAquick gel extraction kit (Qiagen, Hilden, Germany). All five fragments (A, B, C, D, and E) were ligated with T4 ligase (NEB) at 4°C overnight in an equal molar ratio for full-length cDNA. The ligated full-length cDNAs were purified by chloroform extraction and used as templates for *in vitro* transcription using the mMessage mMachine T7 transcription kit (Ambion, Austin, CA, USA). The polyadenylated PEDV N gene transcript was generated using the HiScribe T7 ARCA mRNA kit (NEB) and co-electroporated into the Vero cells with the full-length transcripts at 450 V and 50 µF using a Gene Pulser II electroporator (Bio-Rad, Hercules, CA, USA). At 6–8 h after electroporation, the growth medium was discarded, and Vero cells were cultured in maintenance medium in the presence of 10 µg/mL trypsin. Cells were monitored for CPE following infection. In some cases, CPE was not visible within 3 days, requiring a medium change and extended incubation time before harvesting. Once CPE was observed, the virus was harvested and designated as P0 virus. The P0 virus was then subjected to plaque assay, where five individual plaques per virus were initially picked up and propagated. The whole genomes of well-replicated ones with clear CPE were sequenced by the Linear/PCR Sequencing service at Plasmidsaurus. The clone with the fewest amino acid mutations relative to the designed sequence was selected for further characterization.

### Plaque assay

Monolayers of Vero cells in 6-well plates or 12-well plates were incubated with a 10-fold serially diluted PEDV mutant for 1 h. After that, the inoculum was removed, and cells were washed with PBS. Then cell monolayers were covered with overlay containing 0.5% methyl cellulose or 0.75% agarose in MEM supplemented with 10 μg/mL trypsin and 0.3% TPB (66, 67). At 4 dpi, the cells were fixed with 10% PBS-buffered formalin for 15 minutes and stained with 0.2% crystal violet.

### Growth kinetics for PEDV mutants

Vero or LLC-PK1 cell monolayers in 12-well plates were infected with the corresponding viruses at an MOI of 0.01. After the 1 h adsorption, cell monolayers were washed with PBS (−) to remove those unbound virions and were cultured in maintenance medium. Both cells and supernatants were collected at multiple time points and titrated in 96-well plates for TCID_50_ by the Reed–Muench method (68).

### Interferon sensitivity assay

The sensitivity of PEDV propagation to the human IFN-β pre-treatment in Vero cells was previously validated (55). Vero cells were cultured in medium supplemented with various concentrations of human type I (IFN-β) (R&D Systems, Inc., Minneapolis, MN, USA) for 18 h prior to PEDV inoculation, as previously described (51). After pre-treatment, the medium was removed, and cells were washed three times with PBS (−) before inoculation with each virus at an MOI of 0.01. The infected cells were incubated for an additional 24 h before harvesting supernatants and cells, which were immediately frozen at −80°C. After a single freeze–thaw cycle, the samples were centrifuged, and the supernatants were collected for viral titration by TCID_50_ assay. Interferon sensitivity was calculated as the ratio of the virus titer in IFN pre-treated cells (TCID_50_/mL) to that in untreated control cells (TCID_50_/mL).

### Interferon induction assay

LLC-PK1 cells were inoculated with individual PEDV mutants at an MOI of 1. After the 1 h adsorption, the virus inoculum was removed, and the cells were washed with PBS (−). At 12 hpi, the supernatants were collected to titrate viral infectious titer by TCID_50_, and the total cellular RNA was extracted with TRIzol reagent (Thermo Fisher Scientific). Then, cellular DNA was removed by treating with DNase I (Qiagen). Viral total N gene RNA levels were determined by PEDV-specific reverse transcription-quantitative PCR using the OneStep RT-PCR kit (Qiagen) with forward and reverse primers (forward, 5′-CGCAAAGACTGAACCCACTAAC-3′; reverse, 5′-TTGCCTCTGTTGTTACTTGGAGAT-3′) and a probe (5′-FAM-TGYYACCAYYACCACGACTCCTGC-BHQ-3′) (53). The copy number was determined by standard curves. One microgram of total RNA was reverse transcribed using SuperScript IV Reverse Transcriptase and random hexamers (Thermo Fisher Scientific). The cDNA was subjected to quantitative PCR using SYBR green PCR mix (Thermo Fisher Scientific) according to the manufacturer’s instructions. Primers for IFN detection were described previously (69). The β-actin gene was used as an internal control. The threshold cycle (CT) values for target genes and the differences in their CT values (ΔCT) were determined. Relative transcription levels of target genes are presented as fold changes relative to the mock controls using the 2^−ΔΔCT^ threshold method (70).

### Study of the pathogenesis and immunogenicity of the PEDV mutants in neonatal Gn pigs

Gn pigs were delivered from PEDV-free sows via C-section, as described previously (71). Three to four Gn piglets were housed in one isolator. At 3 days of age, the Gn piglets were orally inoculated with 100 TCID_50_/pig of RMTv1 (*n* = 4), RMTv1-nsp1 + nsp15 (*n* = 5), icPC22A (*n* = 4, as positive control), or MEM (*n* = 4, as mock). In our laboratory, we titrate PEDV using plaque assay for plaque-forming units and/or microwell assay for TCID_50_, and the titers are similar using these two methods for PEDV PC22A. So, this inoculation dose of PEDV corresponds to a value of 100 to 1,000 of the 50% porcine diarrhea dose of the PC22A strain in 4-day-old cesarean-derived, colostrum-deprived piglets (72). One pig from the RMTv1-nsp1 + nsp15 group was euthanized at 4 dpi after it started to shed PEDV RNA in feces for histopathological examinations. One moribund pig from each of the RMTv1 and icPC22A groups was euthanized for histopathological examinations. At 21 dpi, pigs in the RMTv1-nsp1 + 15 (*n* = 4) and mock groups (*n* = 4) were challenged orally with 6 log_10_ TCID_50_ of icPC22A and were monitored for 9 days before euthanasia. This high-challenge dose mimics the infectious dose in the field and was used in our previous PEDV studies with similar ages of pigs (51, 53, 67, 72–74). After virus inoculation, clinical signs, including diarrhea and vomiting, were monitored daily. Rectal swabs were collected daily. The severity of diarrhea was scored based on the fecal consistency in individual pigs: 0, solid; 1, pasty; 2, semiliquid (moderate diarrhea); and 3, liquid (severe diarrhea) (53). The infectious virus titers were determined by microwell infectivity assay for TCID_50_. Viral RNA in the rectal swab samples was extracted using the MagMax RNA isolation kit (Thermo Fisher Scientific) and MagMAX Express instrument (Thermo Fisher Scientific) according to the manufacturer’s instructions, and viral RNA shedding titers were determined targeting the N gene as previously described (53). The copy number was determined by standard curves.

Serum VN antibody titers were measured by TCID_50_-reduction neutralization assay. To prepare the serum-virus mixture, four fold serially diluted serum samples were mixed with an equal volume of icPC22A (100 TCID_50_ of virus/well for the final inoculation). The serum–virus mixtures were incubated at 37°C for 1 h before inoculation of the Vero cell monolayers, with four replicates per dilution. The plates were incubated at 37°C for 4 days. Viral CPEs were observed, and the absence of CPEs indicates that the virus was neutralized. All serum samples were tested at the same time. VN antibody titers were calculated by the Reed–Muench method (68).

### Immunohistochemical staining

The formalin-fixed jejunum and ileum samples were processed using non-biotin polymerized horseradish peroxidase system (BioGenex Laboratories, San Ramon, CA, USA) for IHC staining. Tissues were counterstained with hematoxylin. Monoclonal antibody SD17-103 (gift of Eric Nelson and Steven Lawson, South Dakota State University) targeting PEDV N proteins was used as the primary antibody. Ratios of VH:CD of each group were measured as described before (53). For each intestinal section, 15 villi and crypts were selected and measured from different sections.

### Statistical analysis

The statistical analyses were performed using GraphPad Prism version 10.0. Data in Fig. 2 to 5, 7B, and 8D and Tables 2 and 3 are shown in mean ± standard deviation. Figure 3A and 8D and Table 3 were analyzed by unpaired *t*-tests. Figure 3B, 4A through E, and 7B and Table 2 were analyzed by one-way analysis of variance followed by Tukey’s multiple-comparison test. Groups with significant difference (*P* < 0.05) were indicated with different letters or * (*, *P* < 0.05; ****, *P* < 0.001).

## Data Availability

The data supporting the findings of this study are available within this article and its supplemental material.

## SUPPLEMENTAL MATERIAL

Fig. S1 jvi.00451-25-s0001.tiff Comparison of RMTv1-c2P1 and RMTv1-c2P10 for the effect of 5'-UTR mutations near leader TRS-CS in IFN induction and viral replication.

ASM does not own the copyrights to Supplemental Material that may be linked to, or accessed through, an article. The authors have granted ASM a non-exclusive, world-wide license to publish the Supplemental Material files. Please contact the corresponding author directly for reuse.
