# Supplementary figures and images for: Engineering a recombination-resistant live attenuated vaccine candidate with suppressed interferon antagonists for PEDV
Source: J Virol. 2025 Jun 12;99(7):e00451-25. doi: 10.1128/jvi.00451-25 (PMC12282079; doi:10.1128/jvi.00451-25)

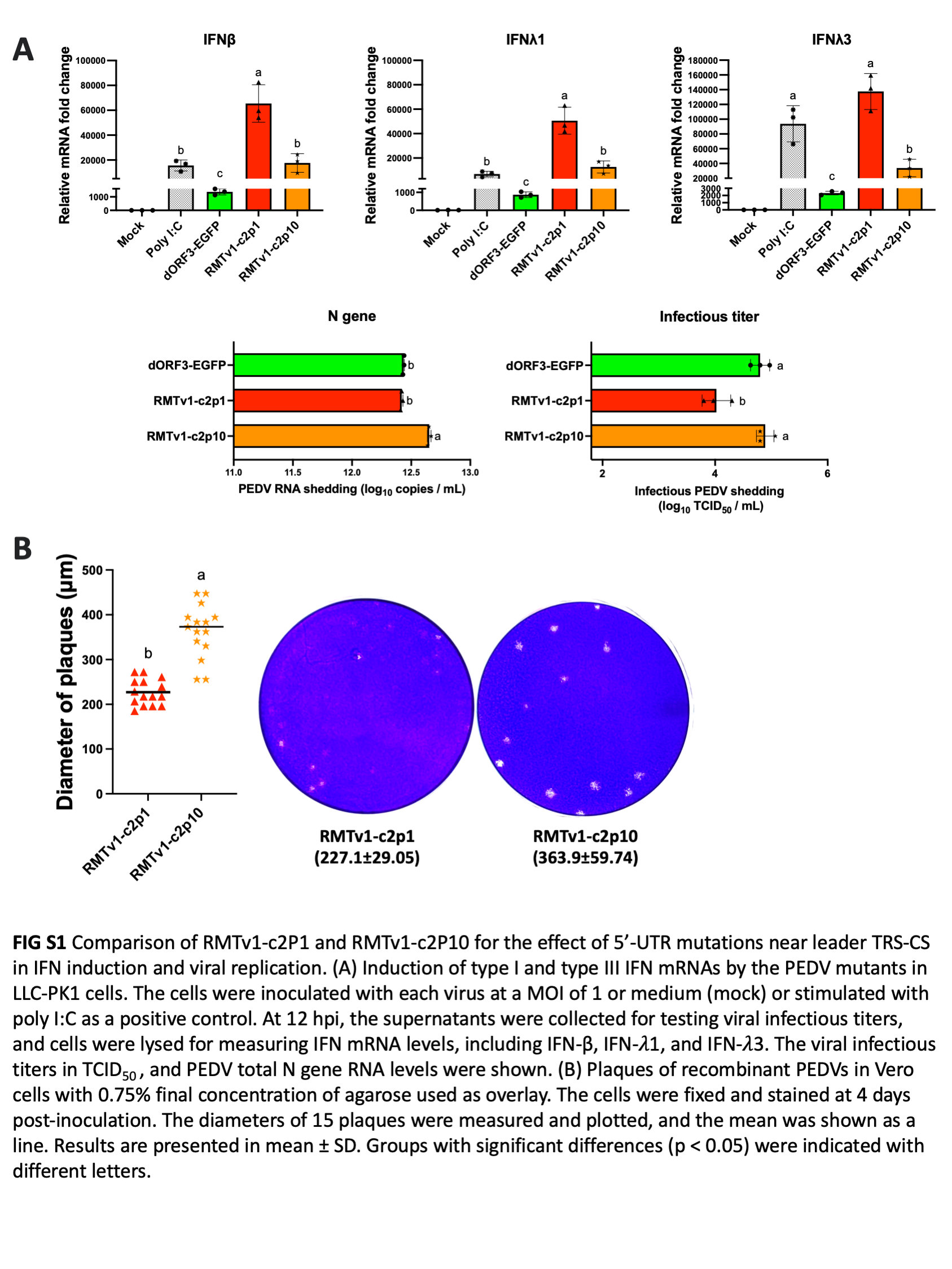

Supplement: Fig. S1 — Comparison of RMTv1-c2P1 and RMTv1-c2P10 for the effect of 5'-UTR mutations near leader TRS-CS in IFN induction and viral replication. [file jvi.00451-25-s0001.tiff]
